# MRI findings in atraumatic shoulder pain—patterns of disease correlated with age and gender

**DOI:** 10.1007/s11845-022-03012-y

**Published:** 2022-05-10

**Authors:** Sarah K. Eustace, Alexandra N. Murphy, Daire J. Hurley, Ahmed H. Alsayegh Abul, Eoin Kavanagh

**Affiliations:** Department of Radiology, National Orthopaedic Hospital, CappaghDublin 11, Finglas Ireland

**Keywords:** Age, Gender, Impingement, MRI, Rotator cuff derangement

## Abstract

**Background:**

The rotator cuff is a group of muscles and tendons which support the shoulder joint. Rotator cuff disease is a frequent cause of morbidity in adulthood.

**Aims:**

The aims of his study are to determine the prevalence and patterns of rotator cuff derangement in symptomatic patients using MRI and to attempt to correlate identified patterns of disease with age and gender

**Methods:**

Five hundred ninety-seven patients attending for MRI of the shoulder with atraumatic shoulder pain were included for study. Patients’ age and gender was recorded. Record was made of the presence or absence of rotator cuff derangement and of degenerative change in the AC and glenohumeral joints. Correlation was made between age and gender.

**Results:**

There were 358 males (60%) and 239 females (40%) with a mean age of 49.4 ± 17.1 years. Subacromial bursitis was identified in 517 patients. A normal supraspinatus tendon was identified in 219 patients and supraspinatus full thickness tearing was identified in 102 patients. A normal AC joint was identified in 267 patients while degenerative AC joint changes were identified in 370 patients. A significant correlation was identified between age and rotator cuff derangement (*p* < .001) and between age and AC joint derangement (*p* < .001). No significant difference was identified between gender and patterns of cuff derangement

**Conclusion:**

The extent of rotator cuff and AC joint derangement increases with ageing. Impingement appears to trigger a cascade of events in sequence, from isolated subacromial bursitis through to supraspinatus tendon tearing. Patterns of rotator cuff derangement are similar in men and women.

## Introduction

Shoulder derangement is a frequent cause of morbidity in adulthood, estimated to affect over 75% of the population over a lifetime [1]. Derangement results in significant debility during activities of daily living and associated positional pain that is a frequently recognised cause of sleep disruption [1]. In most patients presenting with symptoms of shoulder pain and impaired movement, in contrast to other joints, there is no history of an acute traumatic event. In the majority, symptoms develop insidiously and are essentially thought to reflect soft tissue and bony derangement triggered by the rigours of time and repetitive use [2–4].

Attempting to define the cause of symptoms, many have previously reviewed shoulder derangement, combining clinical, surgical, imaging and pathological studies [5–7]. Neer, based on surgical and pathological study, proposed that in most, derangement was triggered by impingement between the acromion and the anterior border of the supraspinatus tendon, resulting in sequential development of subacromial bursitis, supraspinatus tendinopathy and partial and full thickness supraspinatus tendon tearing [8]. Others have postulated that intrinsic changes within the supraspinatus tendon, ischemia and microtearing result in tendinopathy allowing upward migration of the humeral head and secondary impingement progressing as subacromial bursitis and ultimately partial and full thickness tearing [9, 10]. Rarely pain is attributed to intrinsic shoulder impingement between the undersurface of the supraspinatus tendon and the posterior glenoid margin in abduction and elevation or to subcoracoid impingement of the subscapularis tendon with secondary inflammation, although in the majority this accompanies upward elevation of the humeral head in chronic rotator cuff tearing—a manoeuvre that results in narrowing of the subcoracoid space [11].

This study was undertaken to determine the age- and gender-related prevalence of varying forms of shoulder derangement. In doing so, we wanted to ascertain in support of Neer’s hypothesis that atraumatic shoulder derangement of the rotator cuff and acromioclavicular (AC) joint is a progressive predictable event related to ageing and to determine whether patterns of derangement varied in men and women.

## Methods and materials

### Materials

Five hundred ninety-seven patients referred for MRI of the shoulder complaining of atraumatic shoulder pain and morbidity over a 3-year period between January 2017 and January 2020 were included for study. Atraumatic shoulder pain was defined as shoulder pain in the absence of a recognised acute traumatic event. Patients with a clear history of pain triggered by a traumatic event were excluded from the study. Patients were grouped based on age into subgroups: 0–20 years old, 21–30, 31–40, 41–50, 51–60, 61–70, 71–80 and greater than 80 years old. Gender was recorded in each case.

### Methods

Each patient underwent MRI of the shoulder using a dedicated shoulder coil on a 1.5 T Philips MRI. Images were acquired in the oblique coronal, oblique sagittal and axial planes with both STIR (TI 160 ms, TR 2000 ms, TE 20 ms), T1 (TR 500 ms, TE eff 15 ms, TF 4) and T2 (TR 2000 ms, TE eff 80 ms, TF 8) weighting.

### Analysis

Images were reviewed by two musculoskeletal radiologists and findings were recorded independently. Discrepancies in interpretation were resolved by consensus. In each case, record was made of the presence or absence of subacromial bursitis, supraspinatus and rotator cuff tendinopathy, supraspinatus and rotator cuff partial and full thickness tearing and of the presence or absence of AC joint and glenohumeral joint degenerative change.

For the purpose of this study, *subacromial bursitis* was characterised by the presence of fluid signal hyperintensity above the rotator cuff in the region of the subacromial bursa on T2-weighted and STIR coronal oblique images; *supraspinatus tendinopathy* was characterised by localised signal abnormality within the tendon, isointense on T1-weighted images and hyperintense on T2-weighted coronal oblique images; *supraspinatus partial tearing* was defined by observed incomplete tendon discontinuity (superior or inferior surface, anterior margin, posterior margin, enthesial surfaces) observed on T1- and T2-weighted coronal oblique scans; *full thickness supraspinatus tearing* was defined by observed complete full thickness tendon discontinuity in both the antero-posterior and supero-inferior planes on T1- and T2-weighted coronal oblique images; *Acromioclavicular and glenohumeral joint derangement* was defined by evidence of articular surface irregularity, capsular distention, joint space fluid, marginal osteophyte formation and para-articular inflammatory changes into mild, moderate and severe gradings.

Correlation was made between age subgroup, gender and observed findings at imaging using Pearson regression analysis. *P* < 0.05 was considered statistically significant.

### Gold standard

Correlation with history, clinical examination, response to intervention and surgery when undertaken was used as a gold standard.

## Results

**Fig. 1:**
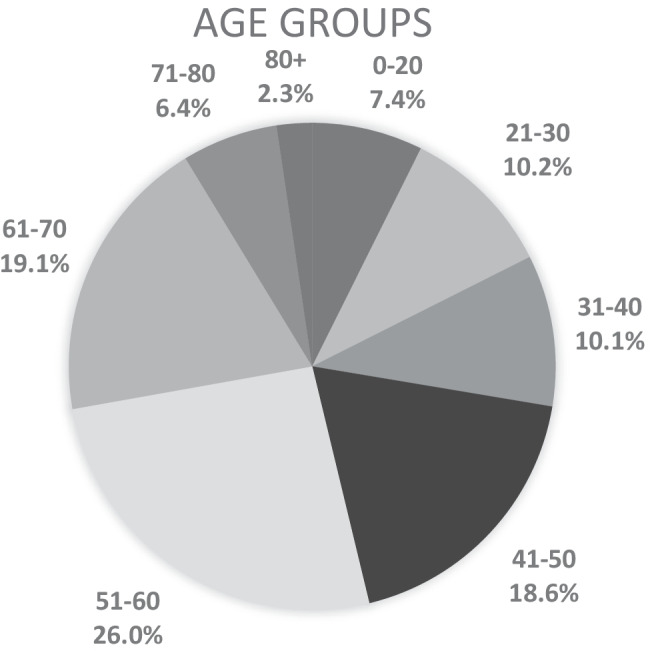
Age groups presenting for MRI of the shoulder with atraumatic shoulder pain

**Fig. 2:**
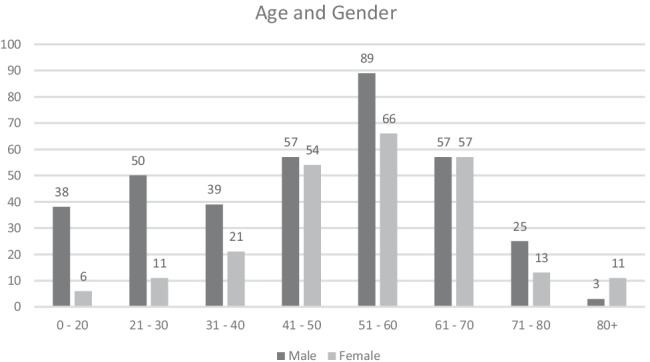
Age and gender breakdown presenting for MRI of the shoulder with atraumatic shoulder pain

**Fig. 3:**
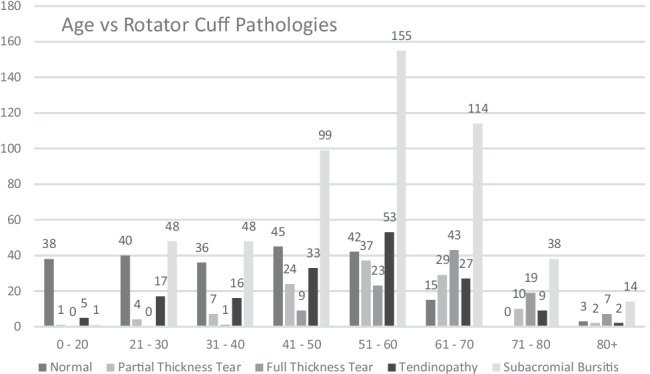
Rotator cuff pathologies subdivided by age in those presenting for MRI of the shoulder with atraumatic pain

Five hundred ninety-seven patients were included for study. There were 358 males (60%) and 239 females (40%). The mean age was 49.4 years (SD 17.1 years) (range 10 to 91 years). Four hundred one (67.1%) right shoulder and 196 (32.9%) left shoulder scans were included for study. Age and gender profile of study subjects is recorded in Figs. 1 and 2. The findings of each rotator cuff pathology subdivided by age are found in Fig. 3.

A *normal supraspinatus* tendon was identified in 219 patients: in 38 of 44 patients (86.3%) between 0 and 20 years, in 76 of 121 patients (62.8%) between 21 and 40 years, in 45 of 111 patients (40.5%) between 41 and 50 years, in 42 of 155 patients (27%) between 51 and 60 years, in 15 of 114 patients (13%) between 61 and 70 years and in only 3 of 52 patients (5.7%) over 70 years.

*Subacromial bursitis* was identified in 517 patients: in 1 of 44 patients (2.2%) between 0 and 20 years, in 98 of 121 patients (80.9%) between 21 and 40, in 99 of 111 patients (89.1%) between 41 and 50 years and in all patients above 50 years of age.

*Supraspinatus tendinopathy* was identified in 162 patients: in 5 of 44 patients (11.3%) between 0 and 20 years, in 33 of 121 patients (27.2%) between 21 and 40 years, in 33 of 111 patients (29.7%) between 41 and 50 years, in 53 of 155 patients (34.1%) between 51 and 60 years, in 27 of 114 patients (23.7%) between 61 and 70 years and in 11 of 52 patients (21.2%) over 70 years.

*Partial thickness supraspinatus* tear was identified in 114 patients: in 1 of 44 patients (2.2%) between 0 and 20 years, in 11 of 121 (9.1%) patients between 21 and 40 years, in 24 of 111 patients (21.6%) between 41 and 50 years, in 37 of 155 patients (23.8%) between 51 and 60 years, in 29 of 114 patients (25.4%) between 61 and 70 years and in 12 of 52 patients (23.1%) over 70 years. It was noted to be at the anterior margin in 79 patients, at the enthesis in 20 patients and the superior or inferior surface in 15 patients.

*Full thickness supraspinatus tearing* was identified in 102 patients: in 0 of 111 patients (0%) up to 30 years, in 1 of 60 patients (1.6%) between 31 and 40 years, in 9 of 111 patients (8.1%) between 41 and 50 years, in 23 of 155 patients (14.8%) between 51 and 60 years, in 43 of 114 patients (37.7%) 61 to 70 years and in 26 of 52 patients (50%) over 70 years.

*Degenerative AC joint changes* were identified in 370 of 597 patients (61.9%): A normal AC joint was identified in 227 of 276 patients (82.2%) up to 50 years. Mild degenerative changes were noted in 141 of 266 patients (53%) between 41 and 60 years, moderate in 155 of 269 patients (57.6%) between 50 and 70 years and severe in 74 of 166 patients (44.6%) above 61 years.

A significant correlation was identified between age and rotator cuff derangement (*p* < 0.001). A significant correlation was also found between age and AC joint degenerative change (*p* < 0.001). However, no significant correlation was identified between gender and rotator cuff or AC joint derangement (*p* > 0.05). Incidental findings included glenohumeral degenerative changes in 38 patients, suprascapular notch ganglion cyst in 2 and biceps tendon rupture in 17 patients all of whom had a full thickness tear of the supraspinatus tendon.

## Discussion

Atraumatic shoulder pain is a common cause of morbidity in adulthood. Studies have suggested that pain is triggered by derangement of the rotator cuff and of the AC joint [12]. Disruption of the glenohumeral joint, biceps tendon injury, inflammatory arthropathy and conditions such as suprascapular notch ganglion cyst and localised myositis (Parsonage Turner Syndrome) are less common [13, 14]. The exact prevalence of shoulder soft tissue derangement is unclear. Existing estimates that up to 50% of the population have full thickness rotator cuff tearing by the age of 65 years are based on clinical, surgical, radiographic, sonographic and pathological studies, limited by indirect assessment or by relatively small study numbers [15–18]. Attempting to correct existing limitations, this study was undertaken to review a large cohort of patients presenting with shoulder pain over a continuous 3-year period based on review of MR images of the shoulder which allow direct visualisation of bone and soft tissue structures.

In this study, 60% of the enrolled patients were male, while 40% were female. This male to female ratio remained similar through all age groups. This male predominance may be explained by gender related differences in muscle bulk, sporting participation and occupational activities [19]. However, when rotator cuff derangement occurs, the results of this study suggest that the pattern of disease in men and women are similar. Although shoulder dominance was not recorded, more patients with right-sided symptoms were included for study. This is likely to be related to right-sided dominance. Worldwide estimates suggest that 90% of the population are right hand dominant, and hence, one would predict that right-sided injuries are more common as in this study [20, 21].

Previously, Neer hypothesised that in most adults, shoulder derangement followed a predictable course initially triggered by the development of impingement within the subacromial space [8]. He proposed that impingement occurred due to positional narrowing of the subacromial space in shoulder abduction and elevation and resulted in irritation of the soft tissues of the subacromial bursa and secondary inflammation or bursitis. He postulated that this primary event initiated a cascade of events leading from supraspinatus tendinopathy through to partial and then full thickness supraspinatus tendon tearing. Although Neer postulated that acquired impingement reflected anatomic variations in the structures of the coraco-acromial arch such as the shape of the acromion, successfully treated by surgical procedures such as acromioplasty [22], others have emphasised the relevance of scapular tilt and motion to maintain the subacromial space during abduction and hence the impact of supporting musculature including the latissimus dorsi, rhomboids and trapezius [16, 23]. Although most authors support Neer’s proposal that soft tissue shoulder derangement follows a predictable pathway [4, 24], others propose that the primary event triggering this pathway is not impingement but the development of supraspinatus tendinopathy or intrasubstance micro-tearing due to the impact of overuse, ischemia and ageing [9, 10]. These authors propose that weakening of the tendon allows elevation of the humeral head leading to acquired secondary subacromial impingement. The fact that many patients with shoulder pain respond to surgical debridement or repair of the tendon in the absence of any attempt to decompress the coraco-acromial arch, such as by acromioplasty, is in support of this premise [9, 10].

In this study, although 4 of 20 patients (20.0%) below 20 years had findings of tendinopathy in the absence of subacromial bursitis, subacromial bursitis in the presence of a normal tendon was identified in 50 of 165 patients (30.3%) up to the age of 40 years. These findings suggest that generally subacromial bursitis precedes the development of supraspinatus tendinopathy, while in a minority of patients, all below 20 years of age, the opposite process occurs. Examples of this can be seen in Images 1 and 2.

The relevance of subacromial bursitis as a cause of shoulder pain, potentially treated by rest, physiotherapy, anti-inflammatories and occasionally image-guided injections, has been addressed by previous authors [25–27]. Similar to clinical experience, this study shows increasing prevalence of imaging features of bursitis with ageing. Indeed, imaging features of subacromial bursitis, often accompanied by degrees of rotator cuff derangement, were identified in the majority of patients over the age of 40 years and in all patients imaged over 50 years of age. The sensitivity of fat-suppressed imaging to fluid in the subacromial space likely overestimates the prevalence of significant bursitis, and in some of the recorded cases, fluid identified within the bursa may have been within normal range, physiological rather than pathological.

Whether triggered by primary or acquired secondary subacromial impingement, the prevalence of co-existent rotator cuff derangement with supraspinatus tendon tearing increased significantly with age. In such a way, tendon abnormality (tendinopathy or partial thickness tendon tearing) was identified in 53% over the age of 50 years and full thickness tearing in 42% of patients over 60 years of age. Examples of partial and full thickness tears identified with MRI can be seen in Images 3, 4 and 5. Similar to rotator cuff derangement, the prevalence of AC joint derangement was shown to increase significantly with ageing. Degenerative changes of some form were identified in all patients over the age of 50 in this study.

This study was not undertaken to define the exact cause of pain in individual patients**.** Previously, authors have indicated that shoulder pain likely reflected subacromial bursitis, partial thickness tearing or degenerative changes in the AC joint [4, 9, 10, 24]. With ageing, patients in this study were shown to have more complex derangement than previously thought and hence the exact cause of symptoms becomes harder to define with multiple pathologies at play simultaneously.

Although the numbers included in this study are large, this study has limitations. Being retrospective, the clinical details of the enrolled subjects were limited to those included in the clinical referral. No control population was included in the study reflecting difficulty imaging a large cohort of asymptomatic patients. Previous studies have specifically reviewed the prevalence, patterns and imaging appearances of rotator cuff derangement in asymptomatic patients [28–34]. In one study of 420 asymptomatic patients, full thickness rotator cuff tears were identified in 7.6% of patients with increasing prevalence with ageing [35]. Features of derangement that tend to produce pain are not clear although one study attempted to address this issue by following a cohort of asymptomatic patients with cuff derangement and then re-assessing them when they became symptomatic. They noted an increase in the size of the rotator cuff tearing being a relevant feature of symptom development [36].

In summary, this study indicates that when MRI of the shoulder is undertaken in patients presenting with atraumatic shoulder pain, the observed findings are closely related to the patient’s age. In patients over the age of 60, full thickness supraspinatus tendon tears were identified in 42%. Additionally, atraumatic shoulder pain is slightly more common in men and not surprisingly more commonly affected the dominant right side. Nevertheless, when comparing findings in men and women, there was no difference in the pattern of derangement and no difference in the age-related progression of derangement when it occurred. In patients with atraumatic shoulder pain, impingement appears to be the primary insult triggering a predictable progression of changes in sequence, subacromial bursitis, supraspinatus tendinopathy, partial tearing through to full thickness supraspinatus tendon tearing. Isolated bursitis identified at onset of symptoms progresses to complex derangement with ageing.
